# Study on the therapeutic potential of induced neural stem cells for Alzheimer's disease in mice

**DOI:** 10.1186/s40659-024-00568-0

**Published:** 2024-11-24

**Authors:** Qiongqiong Ji, Yuanhao Lv, Bei Hu, Yue Su, Imran Ibrahim Shaikh, Xu Zhu

**Affiliations:** 1 Department of Medical Imaging, Shanghai Children’s Hospital, School of Medicine, Shanghai Jiao Tong University, Shanghai, 200062 China; 2 Department of Orthopedic Surgery, The Second Affiliated Hospital of Zunyi Medical University, Zunyi, 563000 Guizhou China; 3 Fuzhou Medical College of Nanchang University, Fuzhou, 344099 Jiangxi China; 4 Central Laboratory of The Lishui Hospital of Wenzhou Medical University, Lishui People’s Hospital, The First Affiliated Hospital of Lishui University, Lishui, 323000 Zhejiang China; 5 Department of Respiratory and Critical Care Medicine, School of Medicine, Shanghai Pulmonary Hospital, Tongji University, Shanghai, 200433 China; 6 Department of Orthopedics, Tongji Hospital, Tongji University School of Medicine, Shanghai, 200065 China

**Keywords:** Alzheimer’s disease (AD), Mouse embryonic fibroblasts (MEFs), Induced neural stem cells (iNSCs), Sox2, Valproic acid (VPA), Transplantation treatment

## Abstract

Induced neural stem cells (iNSCs), which have similar properties to neural stem cells and are able to self-proliferate and differentiate into neural cell lineages, are expected to be potential cells for the treatment of neurodegeneration disease. However, cell therapy based on iNSCs transplantation is limited by the inability to acquire sufficient quantities of iNSCs. Previous studies have found that mouse and human fibroblasts can be directly reprogrammed into iNSCs with a single factor, Sox2. Here, we induced mouse embryonic fibroblasts (MEFs) into iNSCs by combining valproic acid (VPA) with the induction factor Sox2, and the results showed that VPA significantly improved the conversion efficiency of fibroblasts to iNSCs. The iNSCs exhibited typical neurosphere-like structures that can express NSCs markers, such as Sox2, Nestin, Sox1, and Zbtb16, and could differentiate into neurons, astrocytes, and oligodendrocytes in vitro. Subsequently, the iNSCs were stereotactically transplanted into the hippocampus of APP/PS1 double transgenic mice (AD mice). Post-transplantation, the iNSCs showed long-term survival, migrated over long distances, and differentiated into multiple types of functional neurons and glial cells in vivo. Importantly, the cognitive abilities of APP/PS1 mice transplanted with iNSCs exhibited significant functional recovery. These findings suggest that VPA enhances the conversion efficiency of fibroblasts into iNSCs when used in combination with Sox2, and iNSCs hold promise as a potential donor material for transplantation therapy in Alzheimer’s disease.

## Introduction

Alzheimer's disease (AD) represents one of the most significant social, economic, and medical challenges of our time due to its insidious onset, prolonged progression, and unclear pathogenesis [1]. Substantial evidence demonstrates that the irreversible decline in cognitive abilities in AD patients is closely associated with the deterioration and degeneration of neurons in the AD brain [2, 3]. Current therapeutic approaches offer limited effectiveness and fail to compensate for the neuronal loss in the cortex and hippocampus. As a result, the repopulation and regeneration of depleted neuronal circuits through the introduction of exogenous stem cells present a rational therapeutic strategy [4].

Neural stem cells (NSCs) possess the ability to self-renew and differentiate into various cell types within the nervous system [5]. NSCs hold the potential to functionally replace lost neurons, reinforce impaired synaptic networks, and repair the damaged AD brain. Numerous studies have demonstrated that NSCs can survive, migrate, and differentiate into the main neuronal cell types, offering new avenues for potentially treating neurodegenerative diseases like AD [6]. Furthermore, NSC transplantation has been shown to significantly improve cognitive function in transgenic models of AD [7, 8]. However, directly obtaining NSCs from humans is impractical and unethical.

The direct conversion of mouse fibroblasts into induced neural stem cells (iNSCs) offers a new perspective for acquiring patient-specific NSCs [9]. However, the techniques used for this conversion are complex, labor-intensive, and exhibit low reprogramming efficiency. Therefore, further exploration of more efficient transformation methods is necessary. The conversion of somatic cells into iNSCs relies on two primary approaches: direct and indirect reprogramming. Indirect reprogramming involves classic Yamanaka reprogramming factors (RFs) such as OCT4, Sox2, and KLF4 [10–12]. In the direct approach, a transient and unstable intermediate cell population is directed towards a neural stem cell fate by replacing the reprogramming medium with a neural medium containing specific growth factors [13, 14]. Challenges remain in the clinical application of these techniques due to the potential for unintended genetic modifications from the introduction of exogenous genes and the low conversion rate of direct reprogramming [15]. In this study, we demonstrate that mouse embryonic fibroblasts (MEFs) can be directly reprogrammed into iNSCs using a combination of RFs and chemicals. These iNSCs reduce the risk of exogenous genetic mutations and improve the conversion rate to some extent.

While numerous studies have shown that transplanted NSCs can differentiate into neurons and astrocytes, significantly improving the learning and memory deficits in AD animal models [16–19], no studies have yet investigated the therapeutic potential of iNSCs for AD. The present study aims to: (1) determine whether MEFs can be directly reprogrammed into iNSCs using a combination of Sox2 and the chemical A-83-01, and (2) evaluate whether these iNSCs can be expanded in vitro to improve cognitive performance in AD mouse models.

## Materials and methods

### Animals and groups

All mice were bred and housed at the animal facility of Tongji Hospital. All animal procedures adhered to the guidelines of the Animal Ethics Committee of Tongji University (Approval No. T3-HB-LAL-2023-25). Additionally, the study received approval from the Shanghai Ethics Committee, and all experiments were conducted in accordance with the regulations of the Chinese Animal Welfare Agency.10-month-old male APP/PS1 double transgenic mice and wild-type littermates were obtained from Jackson Laboratory (Bar Harbor, ME, US). All mice were divided into five groups randomly: (1) AD Group (APP/PS1 mice received no treatment); (2) AP Group (APP/PS1 mice received PBS injection); (3) AN Group (APP/PS1 mice received iNSCs injection); (4) WT Group (Wild type received no treatment); (5) WN Group (Wt received iNSCs injection).

### Cell culture

Mouse embryonic fibroblasts (MEFs) were isolated from C57 mouse strain embryos at embryonic day 15 after carefully removing the head and all the internal organs, including the spinal cord. MEFs maintained in DMEM (Gibco) containing 10% FBS (Gibco) and 1% penicillin/streptomycin (Gibco). The control NSCs and established iNSCs cultured in NSCs medium: DMEM/F12 supplemented with 2%B27 supplements (Gibco), 1% N2 supplements (Gibco), 20 ng /ml of epidermal growth factor (EGF, Gibco), 20 ng/ml of fibroblast growth factor (bFGF, Gibco), 1%penicillin/streptomycin. For differentiation, cells were kept in a Neurobasal medium (Gibco) supplemented with 1% l-glutamine (100×) and 10% FBS (Gibco).

### Induction and characterization of iNSCs from MEFs

MEFs were extracted from the tissue, which carefully segregated the head and all the internal organs, including the spinal cord of C57 mouse embryos at embryonic day 15. iNSCs were generated from C57 MEFs (at passages 1-3) by lentivirus transduction of the transcription factor Sox2. Glass coverslips were placed in wells of a 24-well culture plate and coated with gelatin or poly-l-ornithine/laminin for 30 min at 37°. MEFs were plated at 7.5 × 10^4^ cells/well into all wells in high-glucose DMEM with 10% FBS. Following all walls are divided into five groups: (1) S group (MEFs transduced with 500 µl of Sox2 lentiviruses for 24 h); (2) SM group (MEFs were then transduced with 500 µl of Sox2 lentiviruses for 24 h after cultured with A-83-01); (3) M group (MEFs cultured with A-83-01); (4) NC group (MEFs transduced with 500 µl of null lentivirus for 24 h); (5) CT group (MEFs cultured in high-glucose DMEM with 10%FBS). The culture medium was replaced entirely every day. 3 days after transduction, the cells were collected with accutase (Gibco) and transferred to a fresh six-well plate. At confluency, cells resuspended in 60-mm dishes with NSCs culture medium for primary neurosphere formation. After culturing for 7 days, spheres were collected by gravity and plated onto poly-l-ornithine/laminin-coated six-well plates for monolayer expansion. At confluency, cells were harvested and resuspended for a second round of neurosphere formation. After three rounds of neurosphere formation, the cells were passaged in monolayer cultures in tissue culture-coated plates in an NSC culture medium.

### Cell transplantation

iNSCs were dissociated with accutase (Gibco), washed, triturated, and filtered through a 70-μm mesh. Cells resuspended in the appropriate volume of ice-cold PBS (about 1 × 10^5^ cells/μl). The mice were anesthetized with sodium pentobarbital (40 mg/kg, i.p.) and placed on a stereotaxic apparatus. Rectal temperature was regulated at 37–38 ℃ using a thermostatically controlled heating blanket.5 μl of either vehicle (PBS)or cell suspension was injected into each side of the hippocampus in 5 min at the following coordinates relative to bregma: AP-2.05, ML ± 1.85, DV-2.50. The syringe was left in place for 5 min to allow diffusion into the surrounding tissue before being slowly withdrawn.

### Immunofluorescence and confocal imaging

The cells were collected and transferred onto laminin/poly-l-ornithine-coated 24-well plates. The cells were fixed with 4% paraformaldehyde for 30 min at room temperature and washed with PBS. Then, it was permeabilized with 10% normal donkey serum containing 0.25%Triton X-100 in PBS(PBS-T) for 1 h at room temperature. The cells were incubated overnight at 4 ℃ with primary antibodies and 3% normal donkey serum in PBS-T. After washing in PBS, cells were incubated at room temperature with a secondary antibody containing 3% normal donkey serum in PBS-T for 1 h. Cells were immunostained with the following primary antibodies: polyclonal mouse anti-Nestin (1:200; Abcam), polyclonal anti-Sox2(1;400; Abcam), monoclonal mouse anti-MAP2 (1:100; Abcam), monoclonal rabbit anti-GFAP (1:300; Abcam), monoclonal rabbit anti-GalC (1:300; Abcam), monoclonal rabbit anti-ChAT(1:200;).Primary antibodies were detected with the following fluorescently tagged secondary antibodies: donkey anti-rabbit and donkey anti-mouse. Coverslips with stained cells were mounted on glass slides in a VectaShield mounting medium that contained DAPI. Stained cells were examined with a Radiance 2000 laser-scanning confocal system mounted on a Nikon Optiphot-2 microscope.

### Western blot analysis

Cells were rinsed three times with cold PBS buffer and then were harvested. After adding the RIPA buffer into the cells, the cells were centrifuged at 12000 rpm for 10 min. The supernatants were collected, and the concentration of protein was determined. Equal amounts of the protein samples were loaded per lane. Next, proteins were separated on 10% sodium dodecyl sulfate–polyacrylamide gel electrophoresis. After electrophoresis, the proteins in the gel were transferred to a polyvinylidene difluoride membrane, and the membranes were blocked in TBST containing 5% nonfat dry milk and probed with suitable primary antibodies overnight. The next morning, the membranes were probed with horseradish peroxidase-conjugated secondary antibodies and incubated for 1 h at room temperature. Bands were visualized by using the enhanced chemiluminescence reagent. Finally, the blots were quantified by ImageJ.

### Gene expression analysis by RT-PCR and qPCR

Total RNA was extracted with TriZol, and 1 µg of total RNA was reverse transcribed into cDNA using the high-capacity cDNA reverse transcription kit (Applied Biosystems) according to the manufacturer's instructions. The expression of each sample was normalized based on its β-actin mRNA content. Oligonucleotide primers used are presented in Table 1. Reactions were run in duplicate, and real-time data were analyzed with Rotor-Gene Real-Time Analysis Software 6.0.

**Table 1:** Sequences of primers for quantitative polymerase chain reaction analysis

Gene	Primer sequences (5'-3')
CHAT	Forward: CTGTGCCCCCTTCTAGAGC
	Reverse: CAAGGTTGGTGTCCCTGG
Sox2	Forward: GGCCGAGTGGAAGGTCATGT
	Reverse: TCCGGGTGTTCCTTCATGTG
Nestin	Forward: TCCTGGTCCTCAGGGGAAGA
	Reverse: TCCACGAGAGATACCACAGG
β-Actin	Forward: GGGAAATCGTGCGTGACAT
	Reverse: TCAGGAGGAGCAATGATCTTG

### Behavioral test

Spatial learning and memory abilities were examined with a Morris water maze (MWM;) according to standard procedures for mice [20, 21]. In this study, MWM was performed 2 weeks before transplantation and 1 month, 2 months, and 3 months after cell transplantation. The MWM (Shanghai Mobile Datum Information Technology Co.) contains a black pool (120 cm in diameter) filled with water (22 ℃ ± 2 ℃). The black pool was equally divided into four quadrants. A circular transparent platform was located in the middle of the target quadrant (TD) and submerged 2 cm below the water surface. The behavioral analysis was recorded by ANY-maze software (Stoelting Company, USA) on a laptop computer.

The MWM test procedure comprised two protocols: the place navigation test and the spatial probe test. The place navigation test assessed the learning ability of the mice in the water maze. The place navigation test included three sessions per day at 9 AM and 3 PM for four consecutive days. Each session includes two training trials, with an interval of 20 min. If the mouse climbed into the platform after a period of swimming and stayed on the platform for more than 5 s, it was considered successful in finding the platform, and the time spent reaching the platform was defined as the escape latency. If the mouse failed to find the platform within 60 s, the escape latency was set at 60 s. In this case, the mouse was led onto the platform and left there for 30 s. The spatial probe test aimed to evaluate the memory retention of the mice was performed on the fifth day. The platform was removed from the pool, and one 60-s probe trial was conducted to record the number of crossings in the area where the platform was initially located and the time spent in the TQ.

### Transmission electron microscopy

Three months after transplantation, mice were anesthetized with 2.5% pentobarbital and perfused transcranial with 50 ml of ice-cold normal saline and 30 ml of ice-cold 4% paraformaldehyde (PFA). Brain tissues were collected and placed in fresh 30% sucrose solution for 48 h to cryoprotect tissues and then sliced into 20-μm coronal sections on a cryostat (LEICA CM1950, Leica, Germany). Morphometric classification of synapses and analysis of ultrastructural parameters were performed as described. Recognizable synaptic standards were at least three synaptic vesicles included in the presynaptic component and identifiable postsynaptic membrane-dense area. With a stereological point counting method, the number of synapses in the electron micrograph was counted. For each group, 150 electron micrographs were selected and magnified by 23,000. Thirty slices per brain were analyzed (n = 5 per group).

### Statistical analysis

Statistical analysis was performed using SPSS 21.0 software (SPSS Inc., Chicago, IL, USA). All data was distributed and expressed as mean value ± S.E.M. Statistical analysis was performed using a one-way or two-way analysis of variance (ANOVA) followed by Dunnett's test to compare the different experimental groups. A value of P < 0.05 is considered to be statistically significant.

## Results

### Generation of iNSCs from mouse fibroblasts

**Fig. 1:**
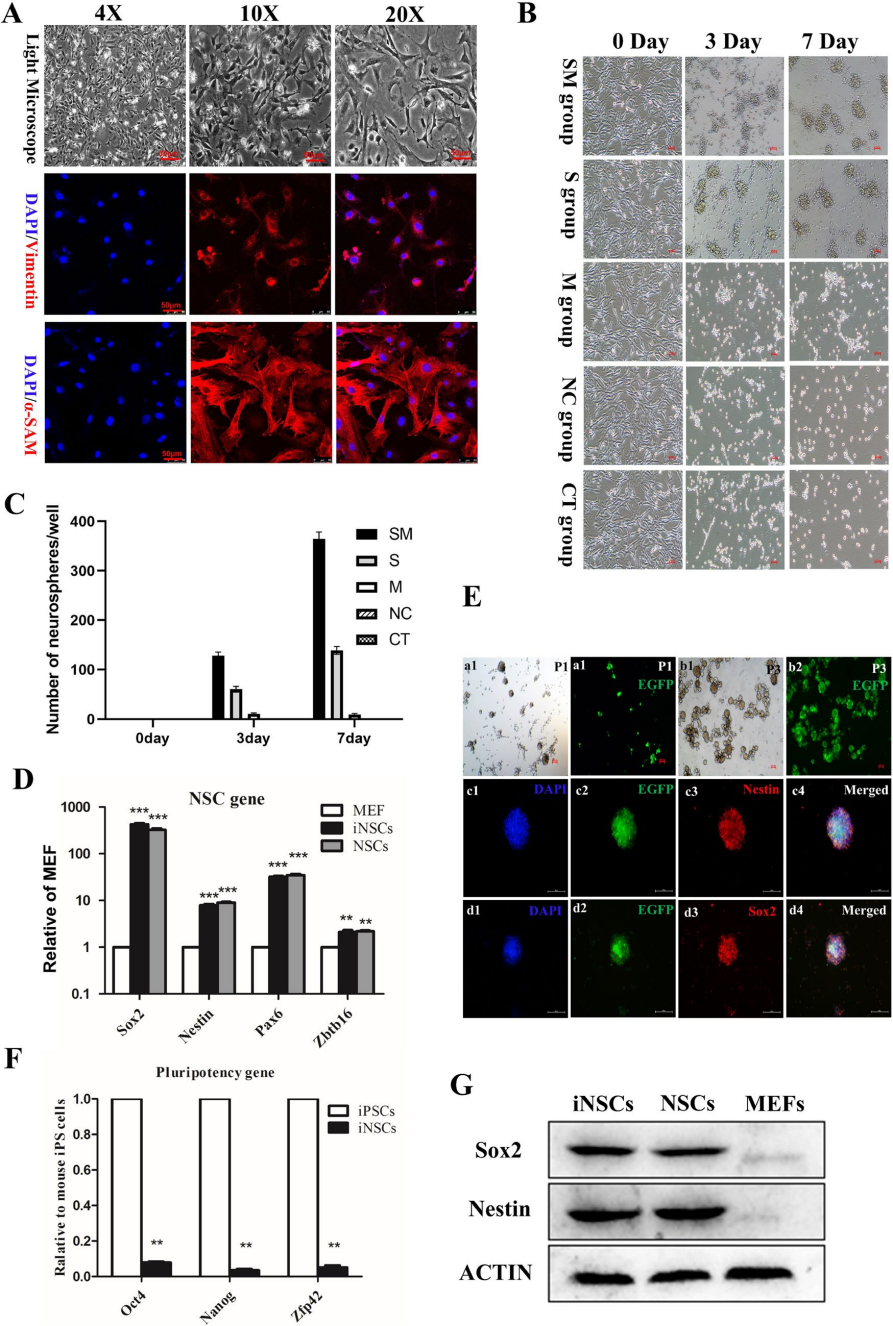
Generation of iNSCs from mouse fibroblasts. **A** Immunofluorescence microscopy images of using Vimentin/α-SMA. **B** The process of cellular transformation: the morphology of the MEFs was changed at 3 days after transfection in the SM, S, and M group; by 7 days after transduction, there were generated neurospheres similar to wild-type NSCs. **C** Quantitative plot of the number of neurospheres. **D** qRT-PCR reveals that iNSCs express typical NSC markers in **D** but do not express pluripotency-related genes **F**. **E** a1-2: the p1 generation of SM group; b1-2: the p3 generation of SM group; The iNSCs expressed NSCs makers, including Sox2(c1-4) and Nestin (d1-4). **G** Expression levels of Sox2 and Nestin in different cells by WB

Numerous studies have demonstrated that induced neural stem cells (iNSCs) can be derived from mouse embryonic fibroblasts (MEFs) using various methods [22, 23]. These iNSCs often exhibit the same morphological and molecular characteristics, including markers such as Sox2 and Nestin, as brain-derived neural stem cells [24]. In this study, we first confirmed the identity of the fibroblasts, with immunofluorescence results showing expression of Vimentin and α-SMA. Given the efficiency and stability of MEF conversion, we selected a single reprogramming factor, Sox2, in combination with A83-01, to induce MEFs into iNSCs (Fig. 1A). To test our hypothesis, we initially cultured MEFs on gelatin-coated plastic in the presence of the small molecule A83-01 to initiate the induction process. We then continued the culture with A83-01 while infecting the MEFs with a lentivirus encoding Sox2. Within 3–4 days post-transduction with Sox2, the morphology of the MEFs began to change significantly, with some transfected cells forming interconnected networks. By seven days post-transduction, neurospheres resembling wild-type NSCs had formed. The number of neurospheres in each well was quantified, as shown in Fig. 1B and C. The number of neurospheres in the Sox2 and A83-01 (SM) group was significantly higher than in the Sox2-only (S) and A83-01-only (M) groups. Additionally, quantitative real-time RT-PCR (qRT-PCR) confirmed that the iNSCs expressed Sox2, Nestin, Sox1, and Zbtb16 (Fig. 1D). Immunofluorescence analysis further demonstrated that the reprogrammed cells were double-positive for Sox2 and Nestin (Fig. 1E) but did not express pluripotency-related genes such as Oct4, Nanog, and Zfp42 (Fig. 1F). In contrast, MEFs cultured in a fibroblast medium for up to four weeks did not show significant expression of Sox2, Nestin, Pax6, and Zbtb16. The protein levels of Sox2 and Nestin in iNSCs were significantly higher compared to MEFs (Fig. 1G).

**Fig. 2:**
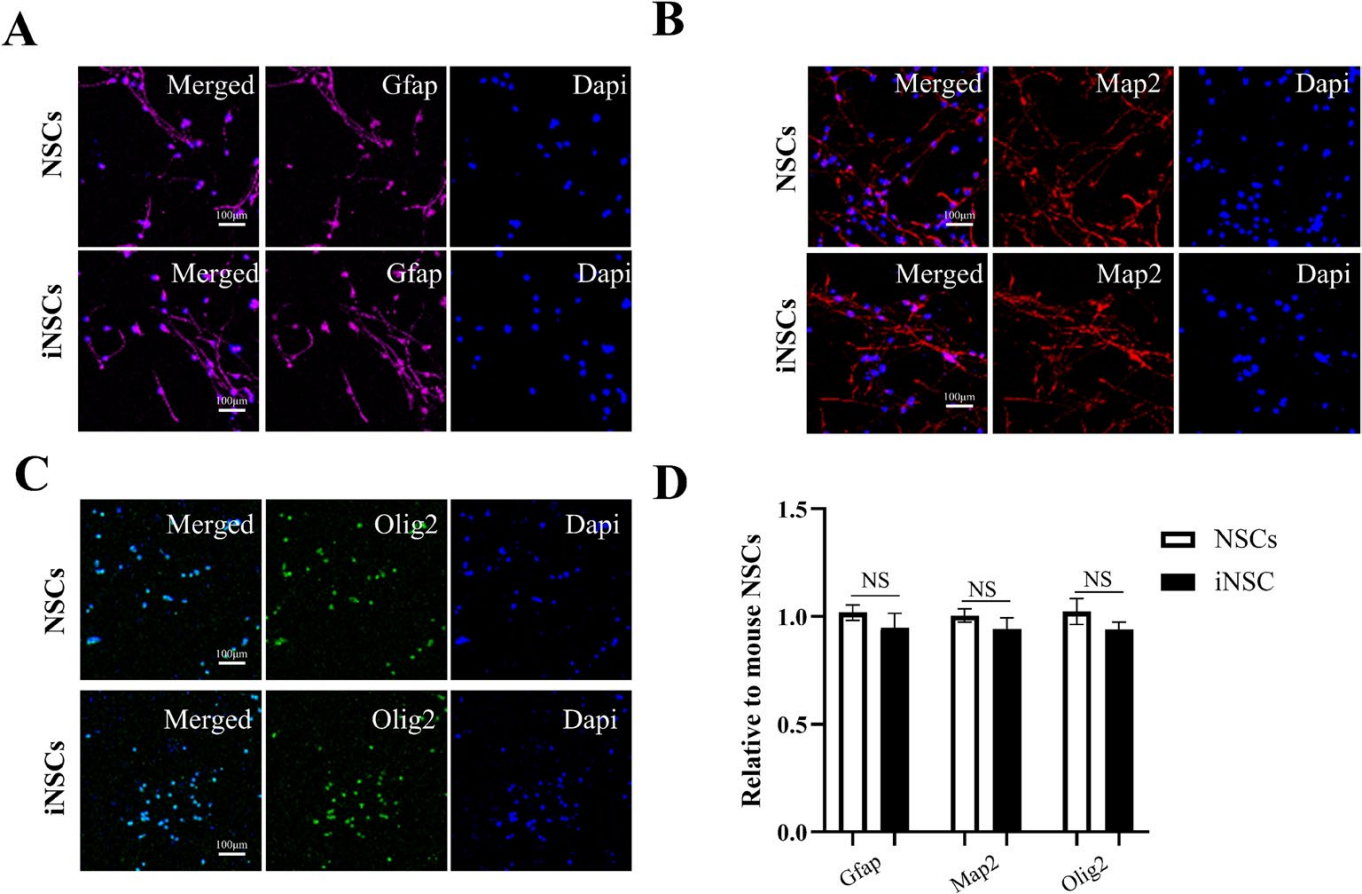
Multipotency of iNSCs in vitro. **A**–**C** iNSCs can differentiate into GFAP + astrocytes, MAP2 + neurons and Olig2 + oligodendrocytes. **D** There was no statistical difference between the proportion of differentiated cells in iNSCs and NSCs

Given the unique nature of the induction process, accurately calculating the induction efficiency was challenging. To address this, we calculated the proportion of Nestin-positive cells among EGFP-positive cells under immunofluorescence microscopy, resulting in an induction efficiency of approximately 3%. This result indicates that the combination of transcription factors and small molecules can enhance reprogramming efficiency. To verify that the established iNSCs possess neural stemness not only at the molecular level but also at the functional cellular level, we evaluated their tripotential differentiation capacity by inducing in vitro differentiation. Under neuronal differentiation conditions supplemented with 1% l-glutamine (100×) and 10% FBS, the ability of iNSCs to generate neurons, astrocytes, and oligodendrocytes was confirmed by immunostaining with antibodies against MAP2, glial fibrillary acidic protein (GFAP), and Olig2, respectively (Fig. 2A–C). Figure 2D shows a histogram demonstrating no statistical difference between the proportion of differentiated cells in iNSCs and NSCs. Taken together, these data suggest that iNSCs acquire neural stemness at the functional cellular level, indicating their potential for therapeutic applications.

### Transplanted iNSCs improve the cognitive ability of AD mice

**Fig. 3:**
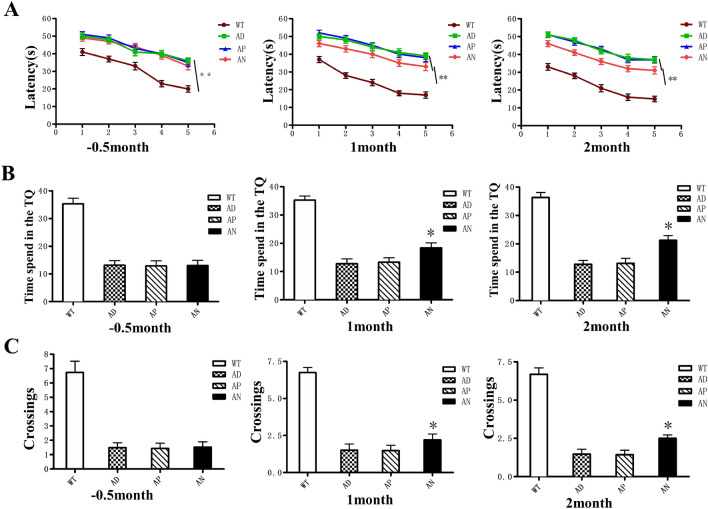
iNSCs transplantation improves cognitive deficits in AD mice. **A** In the place navigation testing of MWM training, AN mice exhibited significantly shorter escape latencies vs. AD mice and AP mice (**B**). In the spatial probe test, AN mice spent a significantly longer time in the target quadrant than AD and AP (n = 10, p < 0.001) and performed similarly to WT mice (n = 10, p > 0.05) as determined by one-way ANOVA. **C** AN mice crossed the platform significantly more than AD and AP mice (n = 10, p < 0.05) and performed similarly to WT mice (n = 10, p > 0.05)

To evaluate whether iNSC transplantation could enhance the cognitive abilities of APP/PS1 mice, we first examined the effects of iNSCs on spatial learning and memory using the Morris Water Maze (MWM) test. Two weeks before transplantation, APP/PS1 mice in all experimental groups took significantly longer to reach the hidden platform compared to the wild-type (Wt) group (p < 0.01), confirming impaired spatial learning in APP/PS1 mice. One month after iNSC transplantation, the latency to find the hidden platform decreased in the mice transplanted with iNSCs compared to those in the AD (untreated) and AP (AD + PBS) groups (p < 0.05 for both), indicating an improvement in spatial learning. However, the learning ability of mice in the AN group (AD + iNSCs) remained slightly lower than that of the WT (wild-type) group and the WN group (Wt + iNSCs), though the differences were not statistically significant (n = 20, p > 0.05). No significant difference was observed between the WT and WN groups (Fig. 3A). In the spatial exploration experiment, the performance of the AN group was comparable to that of the WT group. Mice in the AN group spent significantly more time in the target quadrant (TQ), where the platform was previously located, compared to the AD and AP groups (Fig. 3B), and crossed the location of the original platform more frequently (Fig. 3C) (n = 20, P < 0.001), indicating improved memory retention in APP/PS1 mice. Mice in the WT and WN groups performed similarly (n = 20, P > 0.05). These findings suggest that iNSC transplantation enhances spatial learning and memory in APP/PS1 mice and improves their overall cognitive function.

### iNSCs survive, integrate, and differentiate in vivo without generating tumors

**Fig. 4:**
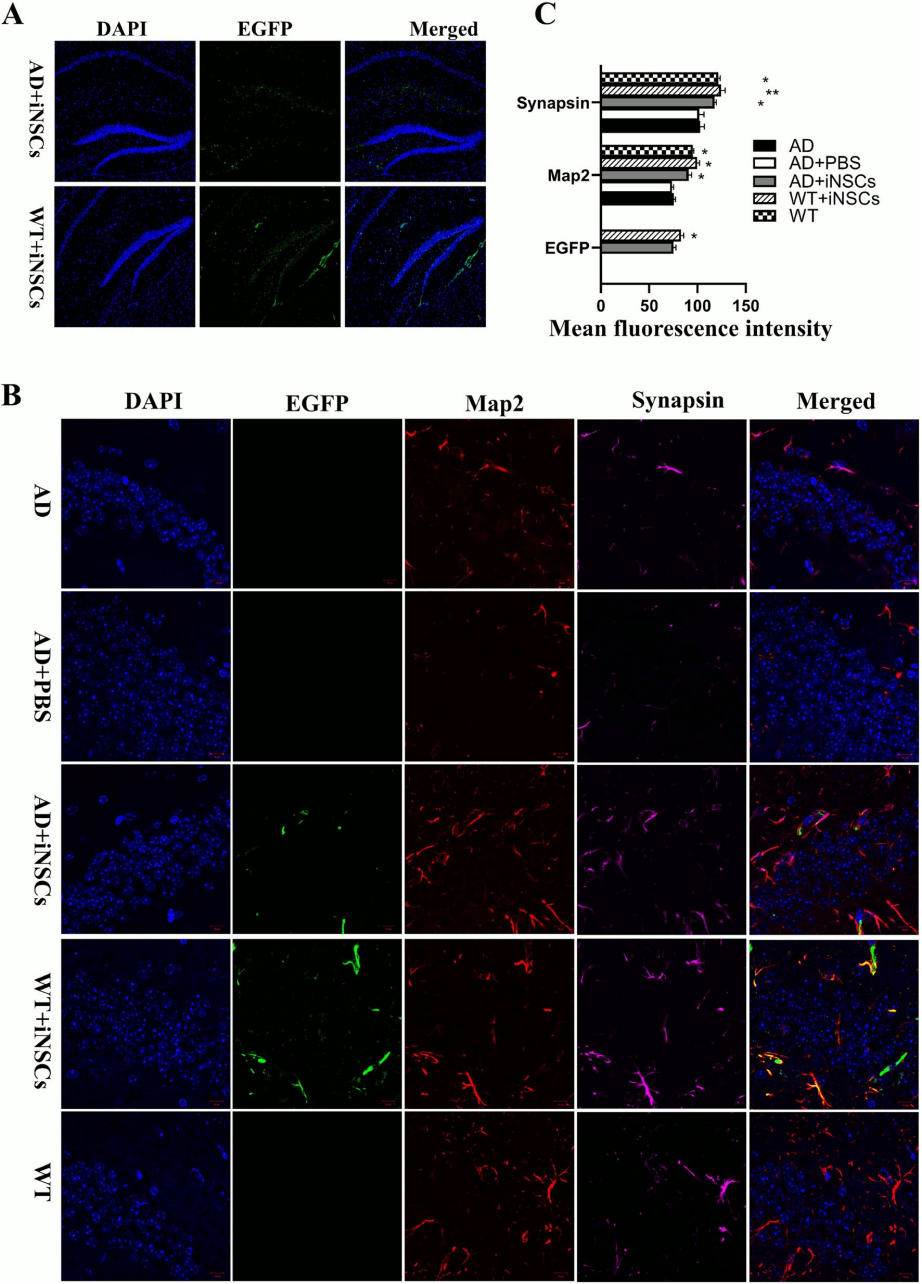
Eight weeks after transplantation, engrafted iNSC had differentiated into neurons, expressing synaptic proteins. **A** EGFP-NSCs (green) are observed in the hippocampal region. iNSCs show higher viability in the brains of WT mice compared to AD model mice. **B** NSCs differentiated into neurons, co-expressing EGFP (green), MAP2 (red), and Synapsin (pink). **C** Histogram showing quantification of fluorescence intensities for EGFP, MAP2, and Synapsin. Data analyzed by one-way ANOVA, *P < 0.05, ***P < 0.01

Our comprehensive analysis of the survival and differentiation status of transplanted iNSCs, conducted 8 weeks post-transplantation, revealed that the cells were able to survive, migrate, and differentiate into three types of neural cells: neurons, astrocytes, and oligodendrocytes. Immunofluorescence results showed a higher density of transplanted cells at the injection site, with a smaller fraction of migrating cells integrating into the existing neural network (Fig. 4A). Importantly, none of the transplanted cells exhibited tumorigenic potential, and there was no significant accumulation of iNSC-derived cells in areas of the brain other than the injection site. This indicates that the transplanted iNSCs did not cause abnormal growth or migration. However, cells in the WN group (wild-type mice receiving iNSC transplants) showed higher viability compared to those in the AN group (APP/PS1 mice receiving iNSCs). Immunofluorescence analysis revealed a statistically significant higher number of EGFP-positive cells in the WN group compared to the AN group at the same magnification (Fig. 4B). These results suggest that iNSCs can survive, integrate, and differentiate in vivo without generating tumors, with better viability observed in wild-type conditions.

### Mechanism of iNSCs transplantation to treat APP/PS1 mice

**Fig. 5:**
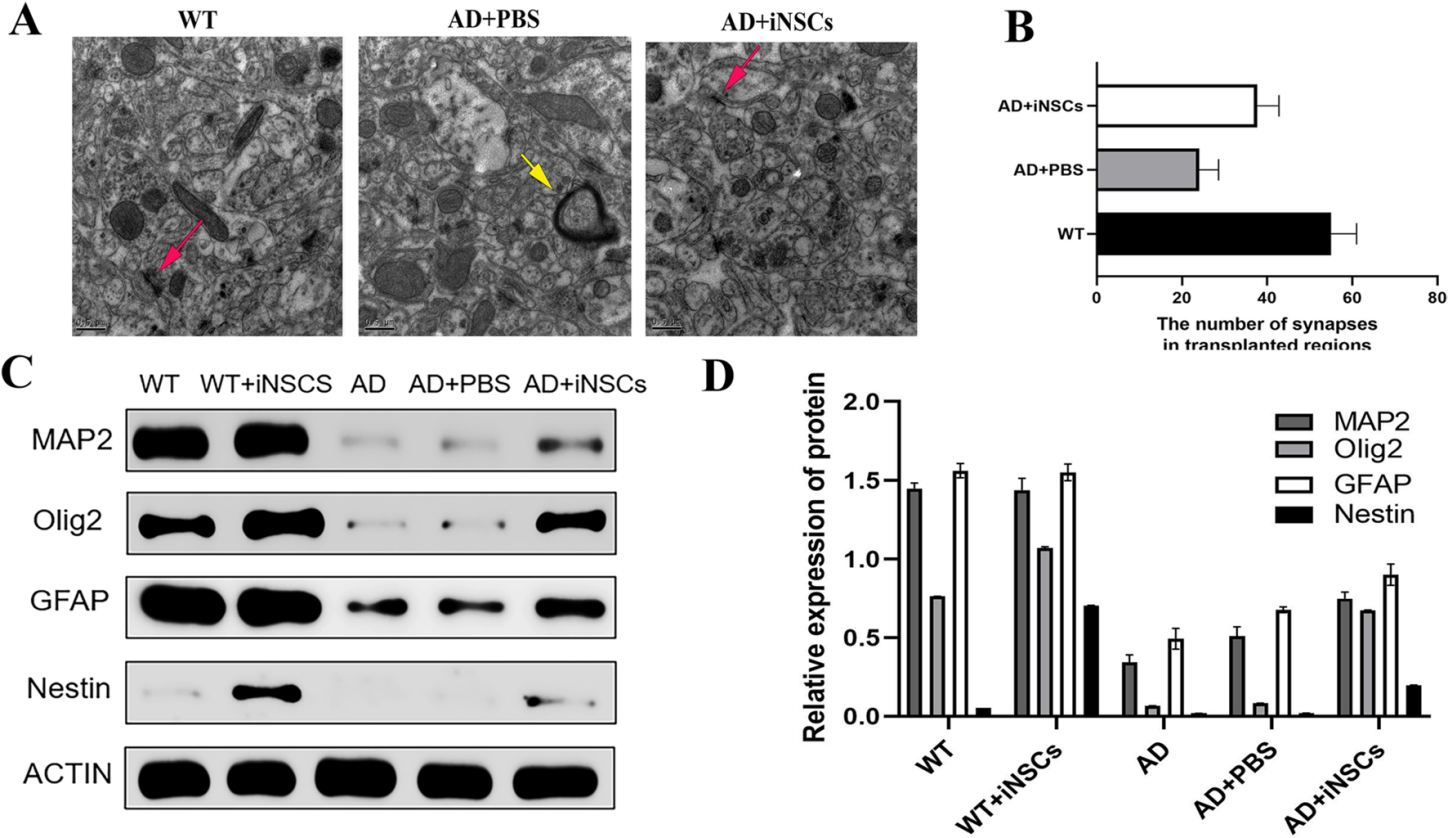
**A** Electron microscopy images showing the ultrastructure of neurons in the transplanted hippocampal region. In both the WT group and AD + iNSCs group, a greater number of synapses (bold arrows) were observed compared to the AD + PBS group. In the AD + PBS group, notable mitochondrial swelling and disrupted cristae structure (thin arrow) were observed within nerve cells. **B** Quantification of the number of synapses in the hippocampal region by EM analysis. AD + iNSCs mice showed a higher number of synapses compared with AD + PBS mice. One-way ANOVA. **C** Typical Western blot of different proteins(Map2, olig2, GFAP and Nestin) in the hippocampal region. **D** Quantification of total protein levels was significantly higher in AD + iNSCs and in WT compared with AD mice and AD + PBS mice. One-way ANOVA, *P < 0.05, ***P < 0.01

The mechanism by which iNSC transplantation benefits APP/PS1 mice requires further investigation. First, we examined whether the transplanted cells could replace the lost neurons in the brain and whether they could form new synapses with existing neurons. Immunofluorescence analysis of synapses in the mouse brain showed that synaptophysin expression was highest in the WN group (wild-type mice with iNSC transplants) and higher in the AN group (APP/PS1 mice with iNSC transplants) than in the AD (untreated) and AP (AD + PBS) groups. This indicates that the transplanted cells were able to form new synapses in the mouse brain (Fig. 4B, C). Next, we compared the ultrastructure of the hippocampus across the different mouse groups using electron microscopy. In the WT and WN groups, hippocampal neurons displayed intact morphology with well-preserved organelles (Fig. 5A, B). In contrast, the AD and AP groups showed significant neuronal damage, including reduced synapses, condensed neuronal nuclei, swollen mitochondria with fewer cristae, and vacuolated mitochondrial matrices. In the AN group, many hippocampal neurons exhibited normal morphology, with abundant, well-structured organelles and intact mitochondrial structures. Additionally, more synapses were observed in the transplanted region compared to the control group. These observations were further confirmed by Western blot (WB) analysis, which was consistent with the electron microscopy results (Fig. 5C, D). Taken together, these data indicate that iNSC transplantation can rescue impaired memory acquisition and recall, a characteristic feature of APP/PS1 mice, by promoting synapse formation and preserving neuronal structure.

## Discussion

Neurons are non-regenerative, which poses significant challenges for therapeutic interventions in neurodegenerative diseases. Neural stem cells (NSCs) hold great promise for improving cognitive function in Alzheimer’s disease (AD) models and are considered valuable for treating AD. However, the difficulty of obtaining NSCs limits the practical application of NSC transplantation therapies, despite their theoretical potential [25]. The transformation of somatic cells into stem cells offers new hope for regenerative medicine. Since the groundbreaking discovery by Takahashi and Yamanaka, the generation of induced pluripotent stem cells (iPSCs) has undergone tremendous advancements. However, clinical application of iPSCs remains problematic due to issues such as directed differentiation to specific cell types and the high tumorigenic potential of iPSCs [26, 27]. The transformation of somatic cells into induced neural stem cells (iNSCs) provides an alternative that bypasses the drawbacks of iPSCs and offers higher clinical potential [28]. In the context of AD treatment, iNSCs offer several advantages. As they are already pre-committed to a neural lineage, iNSCs do not require the lengthy differentiation protocols necessary for iPSCs, making the generation of neurons and glial cells—key elements in AD pathology—more efficient[29]. Moreover, iNSCs present a lower tumorigenic risk, making them more suitable for therapeutic applications aimed at neuroregeneration in AD. There are two main methods for inducing iNSCs: direct and indirect transformation. Direct transformation introduces reprogramming factors to alter the cells' genotype, while indirect methods use small molecules to guide cell differentiation in a targeted direction. Studies have shown that adding reprogramming factors can generate iNSCs with self-renewal capacity and multi-lineage differentiation potential [30].

In this study, we successfully reprogrammed fibroblasts into iNSCs using a combination of small molecules, and the resulting iNSCs were able to stably propagate across generations. However, one limitation of this method is its relatively low transformation efficiency. Therefore, we tried to fuse the two methods to investigate whether the combination of methods could further improve the transformation efficiency of cells and obtain stable iNSCs. Sox2 plays an important role in the cell transformation process, and other studies have shown that the addition of other transformation factors does not improve the transformation efficiency [31, 32]. A-83-01 is a potent TGF-β type I receptor inhibitor, which can inhibit AKL5, AKL4, and AKL7-induced transcriptional processes and improve cell transformation efficiency. Our experimental results demonstrated that the addition of A-83-01 did improve the transformation efficiency of fibroblasts to iNSCs, and the obtained iNSCs had stable self-renewal ability and multi-directional differentiation potential. Despite these promising results, there are limitations to our approach. We did not explore multiple combinations of transcription factors and small molecules in randomized pairings to identify the optimal combination. Nevertheless, our study is among the first to combine small molecules with transcription factors, demonstrating that this approach can enhance cellular transformation efficiency.

We also verified that the iNSCs obtained could survive, migrate, differentiate, and achieve therapeutic effects in vivo, and we selected a mouse model of AD as a therapeutic target. AD is characterized by progressive neurodegeneration, and numerous studies have shown that the development of AD is associated with the loss and damage of neurons in the brain, and NSC have better therapeutic effects on AD [33, 34]. Therefore, we chose the AD mouse model as the treatment target to verify the therapeutic effect of iNSCs. Our results showed that iNSCs transplanted into the mouse brain were able to survive, migrate, and differentiate into neurons, astrocytes, and oligodendrocytes. The microenvironment of AD mice is altered from that of normal healthy mice and to account for the altered microenvironment in AD mice, we included a control group in which iNSCs were transplanted into healthy (wild-type) mice (WN group). This allowed us to assess whether the AD brain environment affected iNSC survival and differentiation. Our results showed that iNSCs had a higher survival rate in the brains of normal mice compared to AD mice, indicating that the AD microenvironment may influence iNSC survival and differentiation. Importantly, we observed no abnormal accumulation of iNSCs in the mouse brains, suggesting that the transplanted cells were non-tumorigenic.

Aβ plaque deposition is a hallmark of AD pathology, and accumulating evidence suggests that Aβ oligomer assembly triggers synaptic loss and hippocampal synaptic dysfunction, which correlates with disease severity [35–37]. Synaptophysin (SYN), a 38-kDa vesicle-associated presynaptic terminal marker rich in dendritic spines, is closely associated with synaptic plasticity and cognitive function. SYN is involved in synaptogenesis, and its level accurately reflects the number and density of synapses, so we chose SYN as an indicator of neuroplasticity. Many studies have shown that SYN is associated with the degree of cognitive decline and the progression of Alzheimer's disease [38]. Significant reductions in SYN have been reported in association with the deposition of Aβ plaques in the hippocampus and cortex, leading to loss of synapses and dysfunction of synaptic transmission [39]. Our findings suggest that iNSC transplantation improved neuronal ultrastructure and rescued synaptic dysfunction in AD mice. Eight weeks after transplantation, the spatial learning and memory deficits seen in AD mice were significantly improved. Increased synaptophysin levels in the transplanted regions suggest that synaptic density may be a key factor in cognitive recovery. Additionally, we observed an increase in both synaptophysin and gap junction protein-43 expression at the gene and protein levels in the transplanted brain regions, suggesting that iNSCs may enhance cognitive function in AD mice by increasing hippocampal synaptic density.

## Conclusion

The combination of the transcription factor Sox2 and the small molecule A-83-01 successfully reprogrammed fibroblasts into iNSCs, significantly improving transformation efficiency. The resulting iNSCs exhibited stable self-renewal and multi-lineage differentiation into neurons, astrocytes, and oligodendrocytes. Importantly, the iNSCs were non-tumorigenic, survived, migrated, and integrated into the brain in vivo, improving the cognitive function of AD mouse models. Thus, iNSCs offer a promising therapeutic strategy for treating neurodegenerative diseases such as Alzheimer's disease.

## Data Availability

The data supporting this study's findings are available from the corresponding authors, YS, II Shaikh, and XZ, upon reasonable request.

## Abbreviations

AD: Alzheimer's disease
NSCs: Neural stem cells
iNSCs: Induced neural stem cells
MEFs: Mouse embryonic fibroblasts
CPV: Combining valproic acid
MAP2: Monoclonal mouse
MVM: Morris water maze
